# Novel Anticancer and Treatment Sensitizing Compounds against Pancreatic Cancer

**DOI:** 10.3390/cancers13122940

**Published:** 2021-06-11

**Authors:** Gabrielle Wishart, Priyanka Gupta, Andrew Nisbet, Eirini Velliou, Giuseppe Schettino

**Affiliations:** 1 Bioprocess and Biochemical Engineering Group (BioProChem), Department of Chemical and Process Engineering, University of Surrey, Guildford GU2 7XH, UK; g.wishart@surrey.ac.uk (G.W.); priyanka.gupta@surrey.ac.uk (P.G.); e.velliou@ucl.ac.uk (E.V.); 2 Department of Physics, University of Surrey, Guildford GU2 7XH, UK; 3 Department of Medical Physics and Biomedical Engineering, University College London, London WC1E 6BT, UK; andrew.nisbet@ucl.ac.uk; 4 Centre for 3D Models of Health and Disease, UCL-Division of Surgery and Interventional Science, Charles Bell House, 43-45 Foley Street, Fitzrovia, London W1W 7TY, UK; 5 National Physical Laboratory, Teddington TW11 0LW, UK

**Keywords:** pancreatic ductal adenocarcinoma, pancreatic cancer, radiotherapy, tumor microenvironment, natural components, anticancer components, chemosensitizer, radiosensitizer

## Abstract

**Simple Summary:**

Pancreatic cancer is a disease of unmet clinical needs. Considering the poor prognosis and high treatment resistance of this devastating disease, the search for the development, understanding and characterization of potential treatments and/or treatment sensitizers is of clinical importance. The synergy between natural remedies and state-of-the-art cancer treatments has been poorly considered despite evidence of antioxidant, antimicrobial and antitumor capabilities. Moreover, natural chemical compounds have been the source of many approved drugs. This review collates novel and natural compounds explored for their preclinical anticancer, chemosensitizing and radiosensitizing effects for pancreatic cancer. Here, we highlight a number of natural sources in very early preclinical testing that may hold potential to enhance treatment sensitization, and in turn, reduce treatment resistance and toxicity via lowering treatment dose requirements.

**Abstract:**

The isolation of chemical compounds from natural origins for medical application has played an important role in modern medicine with a range of novel treatments having emerged from various natural forms over the past decades. Natural compounds have been exploited for their antioxidant, antimicrobial and antitumor capabilities. Specifically, 60% of today’s anticancer drugs originate from natural sources. Moreover, the combination of synthetic and natural treatments has shown applications for (i) reduced side effects, (ii) treatment sensitization and (iii) reduction in treatment resistance. This review aims to collate novel and natural compounds that are being explored for their preclinical anticancer, chemosensitizing and radiosensitizing effects on Pancreatic Ductal Adenocarcinoma (PDAC), which is a lethal disease with current treatments being inefficient and causing serve side effects. Two key points are highlighted by this work: (i) the availability of a range of natural compounds for potentially new therapeutic approaches for PDAC, (ii) potential synergetic impact of natural compounds with advanced chemo- and radio-therapeutic modalities for PDAC.

## 1. Introduction

Natural compound isolation for medical application has been practiced for many decades [1,2]. In particular, the consumption of herbs and spices is associated with antioxidant, antimicrobial and antitumor capabilities [3]. Furthermore, the isolation of chemical compounds for health applications has been sourced from surprising origins. For example, six FDA approved drugs currently exist derived from animal venom for medical use including ACE inhibitors, antiplatelet drugs, thrombin inhibitors and chronic painkillers [2]. A number of naturally sourced plant compounds have also been utilized, such as anticancer agents, e.g., the mandrake plant (*Podophyllum peltatum*) is the original source of podophyllotoxin, the active ingredient in etoposide (VP-16) and teniposide (VM-26) [4]. Moreover, a large range of herbal medicines and natural products have been found to inhibit apoptotic resistance in many cancers via different pathways [5] with 60% of today’s anticancer drugs originating from natural sources [6]. Combination treatments of naturally sourced compounds with state-of-the-art treatment strategies have also been demonstrated to lead to advantageous outcomes such as (i) reduced side effects, (ii) treatment sensitization and (iii) reduction in treatment resistance (via lower treatment dose requirements) [7]. Thus, the search for such natural anticancer compounds is clinically relevant for cancers with high toxicity response profiles to treatments, high treatment resistance and therefore poor prognosis, such as pancreatic cancer.

Pancreatic Ductal Adenocarcinoma (PDAC) challenges global health with increasing incidences and death rates. The late diagnosis and high metastatic occurrence of this disease is associated with non-specific symptoms, labeling this disease a silent killer [8]. Moreover, high levels of treatment resistance elucidate extraordinarily low survival rates that have failed to improve in-line with other cancers. More specifically, PDAC has a 5-year survival rate of just 9%, a 10-year survival rate of 1% and a UK average life expectancy of 4–6 months [9,10]. This 5-year survival rate is extremely low in comparison to other cancer types, i.e., female breast cancer (90%), prostate cancer (98%) and melanoma of the skin (92%) [9]. Five-year survival rates for the majority of common cancers have generally increased since the mid-seventies, e.g., chronic myeloid leukemia saw an increase from 22% to 69% from 2008 through to 2014 [9]. However, PDAC has barely seen an improvement, with this statistic remaining unchanged in over 50 years. An estimated 300,000 cases of PDAC are detected worldwide per year [11] and predictions suggest that PDAC is to be one of the most lethal cancers by the year 2030 [8]. This complex, multigene based disease influenced by many environmental factors, including smoking, is prevalent in European men [9,12,13,14,15].

**Figure 1:**
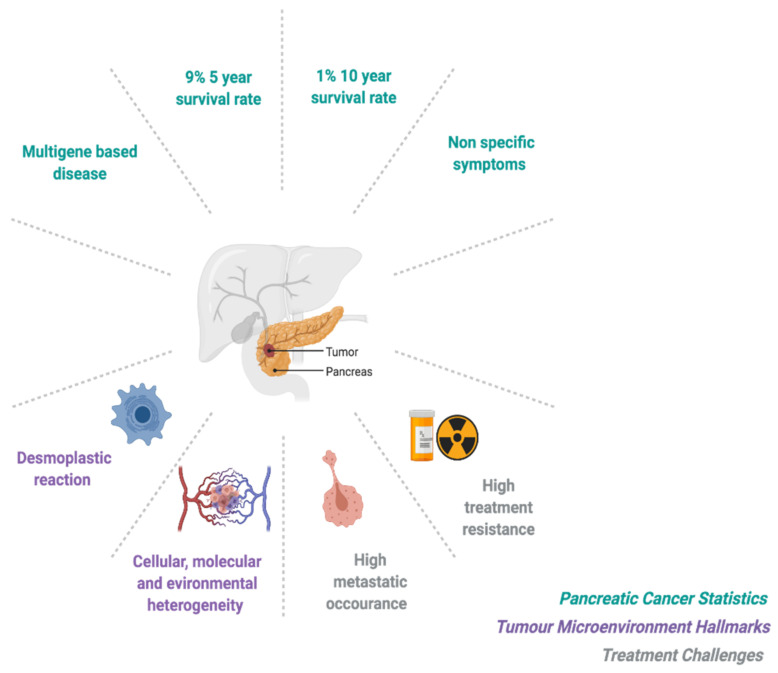
Pancreatic cancer overview: statistics, hallmarks and treatment challenges. Figure created with BioRender.com (accessed on 1 April 2021).

Poor prognosis is associated with advanced disease and high toxicity response profiles significantly effecting treatment efficiency as well as high treatment resistance profiles. Toxicity response for PDAC patients is high due to tumor location, with highly sensitive organs at risk during radiotherapy including the stomach, kidneys, liver and the rapidly dividing cells of the gastrointestinal tract. Furthermore, late onset toxicity reactions can include ulceration, perforation and bleeding as well as, stricture and bowel obstruction due to radiotherapy-induced fibrosis [16]. Chemotherapy for PDAC is often associated with neutropenia, gastrointestinal toxicities including vomiting, diarrhea and oral mucositis [17]. PDAC is notoriously treatment resistance and although the mechanisms of resistance are multifaceted and not fully understood, they are associated with disease specific genetic mutations in oncogenes and tumor-suppressors [5,18,19,20]. Moreover, PDAC encompasses a unique tumor microenvironment (TME) with specific hallmarks that support tumor cell survival and further impair treatment success [11,19,21]. These disease specific hallmarks include tumor cell heterogeneity, dense desmoplasia and hypoxia [19,22,23]. Briefly, pancreatic cancer cell activation of stellate cells stimulates extracellular matrix production, increasing tumor stiffness, known as desmoplasia, in turn this results in the collapse of intra-tumoral blood vessels leading to (i) impaired drug delivery and (ii) heterogeneous expanses of low oxygen, known as hypoxia, that impair radiotherapy efficiency [19,22,23,24]. Figure 1 gives an overview of pancreatic cancer statistics, hallmarks and treatment challenges.

### Treatment for Pancreatic Cancer

Late diagnosis and high metastatic occurrence results in eligibility of just 8–15% of PDAC patients to be considered for curative surgery [10]. Surgery with or without chemotherapy is advised for locally advanced PDAC or for tumors considered to be resectable or borderline resectable [10], i.e., tumors without distant metastasis and venous involvement including superior mesenteric or portal vein and gastroduodenal/hepatic artery [18]. Chemotherapeutics such as gemcitabine and capecitabine are antimetabolites that function as nucleic acid synthesis inhibitors and have been identified as standard and widely used first line therapies for PDAC [25] with combination treatments (GemCap) considered for resected PDAC patients [26]. Erlotinib (a HER1/EGFR tyrosine kinase inhibitor) in combination with gemcitabine has also shown promising potential in improving overall survival [27]. Similarly, nab-paclitaxel (a nanoparticle aluminum bound paclitaxel) treatment in combination with gemcitabine also has shown potential to convert non-resectable disease to surgically resectable disease [28]. The most recently approved chemotherapeutic treatment suggested for consideration for metastatic pancreatic cancer falls under the regime FOLFIRINOX, a combination treatment of four treatments including folinic acid, fluorouracil, irinotecan hydrochloride and oxaliplatin [10], after a landmark clinical trial found that FOLFIRINOX improved overall survival as compared to gemcitabine (i.e., 11.1 months vs. 6.8 months (*p* < 0.0001)) [29].

The American Society of Clinical Oncology advises radiotherapy for local progression or stable disease after a 6-month period of chemotherapy [30]. Chemo-radiotherapy has been a treatment for PDAC option in the US since the GITSG 9173 trial (1985) improved overall survival as compared to chemotherapy alone, i.e., 20 vs. 11 months median survival (*p* = 0.035) [31]. However, more recently two milestone clinical trials resulted in a large amount of uncertainty and doubt surrounding the clinical significance of radiotherapy for pancreatic cancer in Europe. The first of which was the European Study group for Pancreatic Cancer (ESPAC) in which the overall survival of patients treated with chemo-radiotherapy was negatively impacted as compared to chemotherapy alone, i.e., 15.9 vs. 17.9 months medium survival (*p* = 0.05) [32]. It is suggested that deleterious effects on patient prognosis were caused by inconsistent protocol and quality assurance causing substantial toxicity to local organs [33]. The LAP06 Randomized Clinical Trial (2016), resulted in endpoints of no significant difference in patient survival when treated with chemo-radiotherapy as compared to chemotherapy as a mono-therapeutic agent, i.e., 16.5 vs. 15.2 months median survival (*p* = 0.83) [34]. Since these trials, European use of radiotherapy for PDAC has decreased, causing international discrepancy. Thus, it is clear that the best treatment strategy for PDAC is an open and critical debate.

Technical advances in radiotherapy have resulted in a reduction of damage to organs at risk and overall improvement in the survival outcomes for radiotherapy patients. Recent advances include intensity or volumetric modulated radiation therapy (IMRT) or (VMAT), stereotactic body radiation therapy (SRBT) and proton therapy [35,36,37,38,39] These modalities allow for specific tumor targeting, protecting organs at risk, and allow for higher dose escalation [30,35,36,37,38,39]. Moreover, strategies to target hypoxia-induced resistance to radiotherapy have developed over the past few decades. These include hyperbaric oxygen therapy, hyperthermia, carbogen breathing and vasodilators to improve oxygen tension during radiotherapy [40]. Hypoxia Activated Prodrugs (HAPs) and hypoxic nanocarriers are also under investigation to increase radiotherapy efficiency [40,41]. Furthermore, strategies using PARP inhibitors and gene therapy are also being developed to target the complex TME of PDAC and improve radiation effectiveness [14]. However, the failures of radiotherapy sensitizers to reach the clinic are common and multifaceted [40]. This is accentuated by the lack of reproducible and realistic pre-clinical models to encompass TME hallmarks and allow the fast and accurate screening of new compounds and combinatory approaches and improve the translational progression of therapies from bench to bedside.

Despite exciting advances in radiotherapy to protect organs at risk, the clinical requirement for further understanding and development of treatment strategies to combat treatment resistance and lower toxicity profiles for PDAC are still urgently required.

## 2. Natural Anticancer Therapies

**Figure 2:**
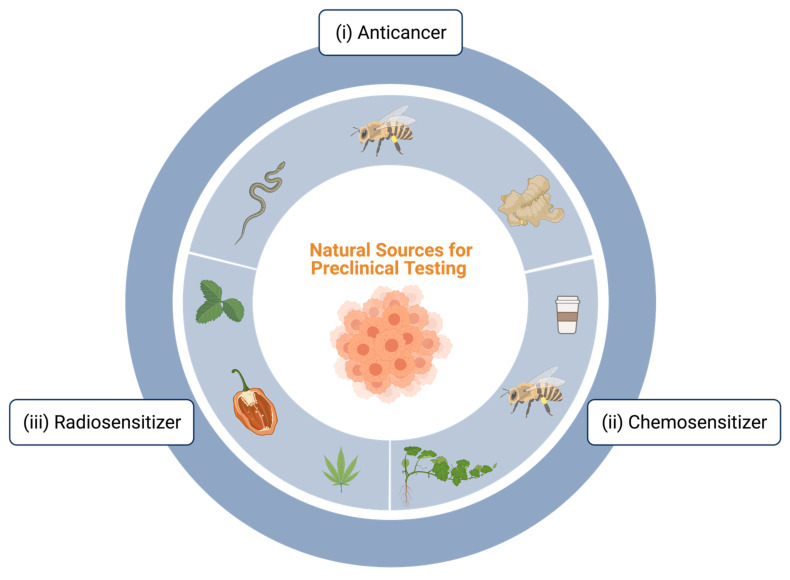
Emerging anticancer and treatment sensitizing compounds from natural sources against pancreatic cancer. Various natural sources have emerged demonstrating (i) anticancer potential in preclinical testing against PDAC cells including, mohave rattlesnake (*Crotalus scutulastus*), prairie rattlesnake (*Crotalus viridis*) and honeybee (*Apes mellifera*) venom, extracts from date palm fruit (*Phoenix dactylifera*), ginger root (*Zingiber officinale*), and cold atmospheric plasma (CAP), (ii) chemosensitizing potential in preclinical testing against PDAC cells including, oridonin isolated from a traditional Chinese medical herb *(Rabdosia rubescens),* trigonelline, a natural constituent of coffee, melittin isolated from honeybee (*Apes mellifera*) venom, curcurbitacin D, a compound isolated from members of the pumpkin and gourd family (Cucurbitaceae), curcumin (*Curcuma longa*) isolated from members of the turmeric and ginger family (Zingiberaceae), and (iii) radiosensitizing potential in preclinical testing against PDAC cells including, curcumin (*Curcuma longa*), neem (*Azadirachta indica*), and raspberry (*Rubus idaeus*) leaf extract, capsaicin, a vanilloid compound, (an active ingredient in hot peppers), and resveratrol, a member of the stilbene family, ascrobate (P-AscH^−^, vitamin C), cannflavin B, a non-cannabinoid, non-psychoactive derivative of *Cannabis sativa L*: FBL-03G. Figure created with BioRender.com (accessed on 1 April 2021).

Natural compounds have been isolated to investigate their potential use as anticancer therapies [5]. More specifically, there is evidence that traditional herbal medicines and natural products may increase treatment success via overcoming apoptotic resistance in pancreatic cancer, decreasing adverse side effects and increase functions of the immune system [5]. Many compounds from natural sources have been tested for their anticancer activity on 2D PDAC cell lines [42]. For example, capsaicin, [43] flavonoids, [44] ursolic and maslinic acid, [45] ginsenosides [46] and many others have demonstrated the ability to induce apoptosis of pancreatic cancer cell lines via numerous pathways associated with oncogene and tumor-suppressor mutations [5]. Herein, we discuss the status of emerging novel and natural pre-clinical research for PDAC including compounds identified for (i) anticancer (Section 2.1), (ii) chemosensitizing (Section 3) and (iii) radiosensitizing (Section 4) activity. An overview of the natural sources discussed in this review is described in Figure 2.

### 2.1. Emerging Natural Anticancer Therapies

Novel and natural anticancer therapies are emerging to target specific characteristics of cancer. Hallmarks of cancer cells include, replicative immortality, evasion of growth suppressors, initiating invasion and metastasis, replicative immortality, initiating angiogenesis and the evasion of apoptosis (programmed cell death) [47]. The induction of apoptosis is the most significant mechanism of many anticancer targets [48]. Apoptotic inducers are emerging from unusual natural origins. More specifically, the isolation of chemical components in certain venoms have shown pre-clinical anticancer potential via apoptosis induction for PDAC cell lines [49,50,51]. Integrins, otherwise known as cellular adhesion receptors for the ECM, are known for their role in cellular adhesion during angiogenesis, invasion and metastasis [52]. Integrin antagonists, known as disintegrins are non-enzymatic, small cysteine-rich proteins with tripeptide motifs (such as RGD), are selective for extracellular matrix integrins such as fibronectin and vitronectin. Disintegrins isolated from snake vemon have been shown to induce apoptosis in the pancreatic cancer cell line BxPC-3 [49]. More specifically, R-mojastin 1, an RGD containing disintegrin cloned from the venom glands of the Mohave rattlesnake (*Crotalus scutulastus*) and R-viridistatin 2 a recombinant disintegrin derived from the Prairie rattlesnake (*Crotalus viridis*) have previously been found to decrease cellular adhesion to laminin and virtonectin, inhibit cellular migration, and induce apoptosis of BxPC-3 cells. Despite this, the disintegrins were three times less potent at inhibiting PDAC proliferation than the chemotherapeutic agent doxorubicin, thus these components are suggested to be adjuvant treatments alongside chemotherapy to help restrain the metastasis of PDAC [49]. These components require further preclinical testing with different cell lines and reliable models to more accurately predict their anticancer potential.

*Apitherapy* involves the use of bee venom as alternative therapies [50]. Melittin is a small peptide component isolated from honeybee (*Apes mellifera*) venom, known to affect the cell membrane, creating pores and contributing to cell lysis [53]. Celik Uzuner et al. were the first to report prolonged cytotoxic effects, via the trypan blue exclusion method, of black sea bee venom. In this research, the pancreatic cancer cell line, AR42J, was treated with 8, 12, 25, 50 and 100 μg/mL of bee venom for 24 h. The number of living cells showed a dose dependent decline for up to 72 h from the removal of the venom [50]. However, this study did not feature chemotherapeutic comparison, nor did they isolate the toxic compound melittin. Melittin (MEL) has also been found to improve the effectiveness of other treatments. More specifically, Shaw et al. (2019) find that the combination treatment of MEL and PT-PBS has a larger cytotoxic effect on the malignant melanoma cell line A375, and breast cancer cell line MCF7 [54]. Moreover, cytotoxicity was quantified via apoptosis/necrosis detection with PT-PBS and MEL alone, i.e., for A375 cells, 38% and 35% apoptosis/necrosis as compared to 96% apoptosis/necrosis after combined exposure (*p* < 0.01), for MCF7 cells, 37% and 30% as compared to 92% apoptosis/necrosis (*p* < 0.01). Furthermore, MTT assay and immunohistochemistry for Ki-67 proliferation marker and tumor weight in ovo supported this synergistic effect as compared to MEL and PT-PBS treatment alone (i.e., MEL and PT-PBS combined treatment reduced tumor weight by 76%, as compared to MEL alone and PT-PBS alone (35% and 30%, respectively)) [54].

*Plasma*, otherwise referred to as the fourth state of matter, is a partially ionized gas. Hot plasmas are ubiquitous in nature, categorized as a high frequency collision of electrons and gas molecules at high temperature, created by arc discharge at equal pressure to that of the atmosphere, they take the form of lightening, flashes, comets and galaxies. Artificial (cold) plasmas are generated in laboratory environments by low frequency or low pressure. The manipulation of this natural source is investigated for their interaction with material via etching, functionalization, cross-linking and deposit formation [55]. Cold atmospheric plasma (CAP) has shown applications for cancer treatment via the production of reactive oxygen (ROS) and nitrogen species production [56,57]. Hattori et al. were the first to investigate antitumor effects of plasma activated media exposure on pancreatic cancer in vitro and in vivo [56]. Briefly, they investigated plasma treatment media effect on that pancreatic cancer cell lines PANC-1, Capan-2, BxPC-3 and MiaPaCa-2 via proliferation assays and apoptosis identification and plasma treatment media subcutaneous injection resulting in mouse models to find a significant reduction in tumor volume as compared to the control group (28 ± 22 vs. 89 ± 38 (mm^3^ ± SD), *p* = 0.0031) [56]. Kumar et al. analyzed the effects of plasma treated water and plasma treated media on the pancreatic cell lines MiaPanCa-1 BxPC-3 and pancreatic stellate cells (hPSC128-SV) to find down regulation of the biomarkers MPK7, BCL2 and CHEK1 responsible for cell proliferation, cell apoptosis and resistance mechanisms [57]. Moreover, this research found an increase in PDAC cell death via apoptosis and necrosis with PTM and PTW treatments, observing stellate cells (responsible for the desmoplastic reaction), to be more resistant to treatment. Furthermore, an upregulation of ROS was identified in both the pancreatic cancer and stellate cells [57]. The application of plasma as an anticancer target for PDAC is very much in its infancy, however, this method may hold potential as a monotherapeutic and or/adjuvant target [58].

The use of neutral argon plasma, otherwise known as *PlasmaJet,* is an emerging technology for cytoreduction (the surgical removal of cancerous cells) for advanced ovarian cancer [59]. Data to support this new technology is limited, with the application also very much in its infancy, however, early data suggest its safety and positive contribution to achieving cytoreduction in advanced ovarian cancer via coagulation and tissue vaporization [59]. This technology can be used in both open and minimal invasive procedures with up-and-coming applications for various surgical procedures [59] and may hold application for other cancer types.

*Flavonoids* are also emerging to target components of the complex and treatment resistant PDAC TME. Al Alwai et al. identify date palm fruit extract to significantly reduce fibrosis of pancreatic stellate cells (PSCs) [4]. As previously mentioned, PSCs play a large role in PDAC progression, metastasis and treatment resistance via the activation of the desmoplastic reaction. Al Alwai et al. activated PSCs and exposed them to nine fractions of ethyl acetate extracted from *Khalas* date fruit for 24 h. The solvent extracts included ethanol, acetone and ethyl acetate significantly suppressed cell proliferation, i.e., *p* < 0.001. As a result of hindering PSC production, these extractions reduced fibrosis/reversed the fibrotic phenotype of PSC cells, identifying this component as a potential anticancer agent, which may have applications for improving treatments that are inhibited by the fibrotic reaction such as PDAC [4].

Natural anticancer agents have also been suggested for use in combination with other treatments. Prescott et al. identified ginger root extract (*Zingiber officinale Roscoe*) and sanguinarine (isolated from the bloodroot plant *Sanguinaria canadensis*) can act as a sono-sensitizer in sonodynamic therapy (SDT) of PANC-1 cells [42]. SDT is an emerging application for ultrasound in the cancer treatment field for its ability to increase permeability of cell membranes increasing drug uptake to specific tissue areas [60]. Prescott et al. found that both compounds initiated dose dependent cytotoxicity, inferring potential anticancer activity. Furthermore, six ultrasound power-frequency configurations revealed sono-mechanical effects of cavitation induced cell death. Combination treatment of 100 μM of sanguinarine and 500 kHz, 10 W revealed a 6% increase of PANC-1 cell death 24 h after exposure, and combination treatment of 1 mM of ginger root extract and 500 kHz, 10 W revealed a 17% increase of PANC-1 cell death of 24 h after exposure [42]. Overall, this research suggests natural anticancer agents such as ginger root extract and sanguinarine may be enhanced by an increased uptake of sonodynamic therapy with a possible synergistic effect, although further research is required to identify the mechanisms at play. Natural and novel anticancer targeting compounds for PDAC are discussed on Table 1. Furthermore, natural and novel anticancer in combination with chemotherapies are discussed in the following section.

### 2.2. Natural Chemosensitsers

The use of gemcitabine is a universally accepted treatment for locally advanced and metastatic PDAC (Figure 2) since the 1990s. Combination treatment of chemotherapy is a widely utilized tool for cancer treatment in general. The advantages of combinatory chemotherapy treatments include drug synergy, increased efficacy and the lowering of individual drug dose, which may result in lower therapeutic resistance [5] and reduce patient toxicity profiles. However, combination treatment attempts for PDAC have generally not been greatly successful [5]. Thus, the investigation of natural compounds as combination treatments and/or adjuvant therapies is an interesting area of research that may hold potential to reduce resistance and/or allow for reduce doses to improve toxicity/patient side effects. Previous research has demonstrated apoptotic enhancement and therefore increased chemo-sensitivity of natural products via MAPK and Nrf2 pathways in PDAC [61,62]. These are specific pathways of interest for PDAC due to their association with genetic mutation of tumor suppressors and oncogenes. Bu et al. reported *Oridonin*, a diterpenoid isolated from *Rabdosia rubescens,* (40 mg/kg) significantly reduced tumor growth in nude mice models when administrated alone as compared to a control (*p* < 0.05). Moreover, gemcitabine alone was as effective as oridonin (*p* < 0.05) and combination treatment resulted in tumor volume reduction as compared to gemcitabine and oridonin treatments alone (*p* < 0.05). This research showed this effect was associated with the up-regulation of the MAPK pathways p38 and p53, finding that oridonin (*p* < 0.05) and gemcitabine (*p* < 0.05) alone significantly up-regulated these pathways, and were even more effective in combination as compared to alone (*p* < 0.05) at inducing cell cycle arrest and apoptosis in pancreatic cancer cells [61]. Furthermore, Arlt et al. investigated the combination of Etoposide and trigonelline, a natural constituent of coffee, to find the inhibition of Nrf2 activity, resulting in apoptotic sensitivity in vitro and in vivo on pancreatic cancer cell lines MiaPacCa2, PANC-1 and Colo357 [62]. More specifically, trigonelline demonstrated the ability to inhibit Nrf2 activity in all cell lines at submaximal doses (0.1–1 µm). Colo357 and PANC-1 bearing mice subjected to combination treatment with Etoposide and trigonelline demonstrated significantly lower tumor sizes as compared to those treated with etoposide alone (*p* < 0.05).

More recently, Wang et al. investigate the cytotoxic effects of melittin (as previously mentioned melittin is a small peptide component isolated from honeybee venom, known to disrupt the cell membrane, creating pores and contributing to cell lysis [53] finding the suppression of tumor growth and promotion of apoptosis as a mono-therapeutic agent, and melittin exposure related gemcitabine sensitization via the cholesterol pathway gene clusterin9 [63]. More specifically, in vitro cell lines were exposed to 1–5 µg/mL for 72 h and assessed via the cell proliferation, colony formation assay and western blot assay to reveal inhibition of proliferation (IC50 value for growth inhibition for SW1990, CAPAN1, AsPC-1 and BxPC-3 was 3.9, 3.4, 2.4 and 1.7 µg/mL), increased apoptosis, induced cell cycle arrest and down regulation of cell cycle related proteins CDK2 and CDK6. Microarray analysis of cell lines exposed to 3 μg/mL melittin for 24, 48 and 72 h revealed the significant suppression of cholesterol biosynthetic process, cellular response to hypoxia and regulation of apoptotic processes (563 differently expressed genes including 394 upregulated and 16 downregulated genes, as compared to control group) [63]. Moreover, knockdown and downregulation of CLU (a molecule of the cholesterol biosynthesis pathway) inhibited SW1990 and CAPAN1 cell growth and inhibited NF-KB/BCL2/P-ERK signaling. Furthermore in vitro, combination treatment of 3 μg/mL melittin for 24, 48 and 72 h and 30 nM gemcitabine on the cell line SW1990 and 2 μg/mL melittin for 24, 48 and 72 h and 10 nM gemcitabine on the cell line Capan1 revealed synergistically reduced cell proliferation via the clonogenic assay and mediated gemcitabine sensitization via CLU expression. These findings have also been supported by in vitro investigations [63].

Sikander et al. investigated curcurbitacin D, a compound isolated from members of the pumpkin and gourd family Cucurbitaceae, to find the inhibition of the expression of key proteins involved in chemo-resistance in the pancreatic cancer cell lines AsPC-1, BxPC-3, CaPan-1 and HPFA-11 [64]. More specifically, pancreatic cancer cell lines were exposed to 0.1–0.5 μM of curcurbitacin D for 24–96 h, revealing dose dependent colony formation inhibition and G2/M growth arrest (at 24 h). The Boyden chamber assay revealed inhibition of migratory potential, a Matrigel invasion assay revealed a suppression of cell invasion and the inhibition of MUC13 expression (a tumorigenic marker) in Curcurbitacin D presence. Combination treatments with gemcitabine revealed also dose dependent inhibition of gemcitabine resistant AsPC-1 proliferation in curcurbitacin D presence. Furthermore, in the presence of 0.5 μM curcurbitacin D the expression of resistance markers (RRM1 and RRM2) assessed via qRT-PCR were significantly reduced as compared to 0 μM (*p* < 0.05). Moreover, in vitro studies indicated inhibition of tumor growth and MUC13 expression supporting anticancer activity of curcurbitacin D [64].

Yoshida et al. investigated gemcitabine resistant PDAC cell lines to find chemo-sensitizing effects of curcumin (isolated from the Zingiberaceae family, a component of *Curcuma longa*, otherwise known as turmeric) [65]. More specifically, gemcitabine resistant cell lines BxPC3, MiaPaCa2 and PANC-1 were seeded in 2D and incubated for 72 h with curcumin (10, 20 and 30 μm) and/or gemcitabine (25, 50 and 75 μm). Colony formation, apoptosis and cell cycle assays revealed combination treatment enhanced BxPC3-GemR cellular cytotoxicity. RNA qRT-PCR analysis revealed curcumin-induced suppression of the proliferative maker PCNA and the tumor suppressor marker p21 suggesting curcumin influence on cell cycle regulation. Western blotting also suggested curcumin treatment suppression of PRC2 (a regulator of cancer stem cells) subunits in all cell lines as well as upregulation of PVT1 (an oncogenic 1ncRNA), and suppression of MYC (regulators of proto-oncogenes) in BxPC-3GemR. These results point towards curcumin sensitivity of gemcitabine resistant cell lines via regulation of PRC2-PVT1-c-Myc axis. This was confirmed by the inhibited development of spheroid-derived cancer stem cell formation. Investigations using xenograft models injected with curcumin and/or gemcitabine for 28 days, revealed reduced tumor growth and inhibited proliferation after combination treatment of curcumin and gemcitabine. Further analysis of these BxPC3-GemR tumors found downregulation of PVT1, Myc and MDR1 expression, suggesting that curcumin re-sensitizes already resistant gemcitabine tumors via PVT1 [65].

Overall, a few natural chemo-sensitizers have been investigated for their ability to sensitize PDAC cell response treatment of gemcitabine and etoposide. These pre-clinical data are very much in their infancy, replication is required in reliable systems to support these findings before application to the clinic can be explored. These natural chemo-sensitisers for PDAC are discussed on Table 2. Furthermore, natural and novel anticancer in combination with radiation are discussed in the following Section 2.3.

### 2.3. Natural Radiosensistisers

Radiosensitizers are greatly needed to address the radio-resistance issue [66] and increase the use and efficacy of radiotherapy approaches for PDAC. Moreover, adverse toxic side effects have preciously caused undesired clinical outcomes for PDAC [32]. Natural radiosensitizers are considered to be safer than synthetic alternatives [66]. The use of natural products to enhance radiation treatment has previously been investigated in various other cancer cells. For example, bioactive food components (BFCs) such as curcumin (turmeric source), genistein (soybean source) and quercetin (ubiquitous source) have previously been investigated for treatment sensitising effects targeting the Nrf2, Akt and Erk pathways [66] and antioxidant activity [67] with some effectiveness in early pre-clinical studies [67]. Sarkaria et al. investigated caffeine as a radiosensitizer for lung cancer cell line A549 and erythroblastoid leukemia cell line K562 [68] and the combination of curcumin and cisplatin has been found to inhibit cell proliferation, invasion and migration [69]. More recently, Bellini et al. studied the application of ginger as a combination treatment with radiation, finding anticancer and radiosensitizing activity in the prostate cell line LN-Cap [70]. Furthermore, flavonoids show potential for radiosensitization in the human cervix cancer cell line HeLa, breast cancer cell line MCF-7 and human colorectal cancer cell line DLD1 in vitro and in vivo [71]. Zhu et al. were the first to show melittin induced radio-sensitivity in both 2D and xenograft models of esophageal cancer [72]. However, the prevalence of pre-clinical radiosensitizers for PDAC is less common in the literature as compared to other cell lines or to chemosensitizers.

BFCs are beginning to emerge as radiosensitizers for PDAC. Veeraraghavan et al. identified the radiosensitizing potential of *Curcumin longa* root extract, neem leaf extract, and raspberry extract [73]. More specifically, BxPC-3, MiaPaCa-2 and PANC-1 cell lines were exposed to single high dose (SDR) (10 Gy) or fractioned radiation (FR) (2 Gy/d for 5 days), to find inhibition of SDR and FR induced genes. All three extracts conferred radiation inhibited cell survival, apoptosis and NF-κB expression [73]. More recently, Schwarz et al. also investigated the radiosensitivity of pancreatic cancer cells lines to Curcumin longa root extract [74]. The pancreatic cancer cell lines PANC-1 and MiaPaCa-2 clonogenic survival fraction was reduced to 50% when treated with a 9.5 and 9 μm of Curcumin. PANC-1 cells were radiosensitized after 24 h of incubation with 10 (4 Gy: *p* = 0.0048, 6 Gy: *p* = 0.0096) or 12 μm of Curcumin (4 Gy: *p* = 0.0028, 6 Gy *p* = 0.0003, 8 Gy: *p* = 0.00070) whereas MiaPaCa-2 cells showed no significant radiosensitization. Furthermore, curcumin increased radiation induced apoptosis in both cells lines after 8 Gy exposure (PANC-1: *p* = 0.0174, MiaPaCa-2: *p* = 0.0043) [74]. This research also identified curcumin induced DNA damage via γ-H2AX evaluation and radiation induced G2/M arrest [74].

Further investigation of BFCs such as capsaicin, a vanilloid compound, (an active ingredient in hot peppers), and resveratrol, a member of the stilbene family, (a polyphenol non-flavonoid compound) also indicated induced radiosensitivity on pancreatic cancer cells. More specifically, Vendrely et al. report capsaicin and resveratrol combination increased sensitivity to radiation in the pancreatic cancer cell line Capan-2 (6 Gy X-ray) as compared to BFCs or radiation alone (*p* < 0.001), however they also reported toxicity but no radiosensitisation in the cell line PANC-1 in vitro [75]. Furthermore, capsaicin and resveratrol significantly increased ROS production after 6 Gy X-ray combination treatment as compared to BFC or radiotherapy alone (*p* < 0.01) in the pancreatic cancer cell lines Capan-2 and BXPC-3. This research also reported measurements on Capan-2 xenografts, revealing lower tumor progression and higher effects of combination treatment including stagnation of tumor growth resulting in a 40% difference in tumor volume as compared to radiation alone (*p* = 0.006 and *p* = 0.001). Furthermore, western blotting of Capan-2 xenograft tumors revealed the inhibition of γ-H2AX in radiotherapy and BFC combinations as compared to control (*p* < 0.05), and further downstream ratios of BAX/BCL2 proteins significantly increased in combination treatments as compared to radiation treatment alone (*p* < 0.05) [75].

Alexander et al. investigate pharmacological ascrobate (P-AscH^−^, vitamin C) as a radio-sensitiser for PDAC [76]. More specifically, MiaPaCa-2 showed decreased clonogenic survival when treated with 1 mM (10 pmol cell^−1^) P-AscH^−^ or 1 Gy alone, and when given in combination as compared to P-AscH^−^ or 1 Gy radiation alone (*p* < 0.05). Furthermore, this research showed increased recovery of clonogenic survival capability in healthy cells (foetal human intestinal epithelial cell line FHs 74 Int) when irradiated in the presence of P-AscH^−^ as compared to radiation alone (*p* < 0.05) suggesting an overall radio-protective effects of P-AscH^−^. Briefly, DNA damage quantification (γH2AX via PCR) revealed an increase in the frequency of DNA lesions in MiaPaCa-2 cells in combination treatment (5 mM P-AscH^−^ and 5 Gy radiotherapy) (0.31 ± 0.05 lesions/10 kb) as compared to radiotherapy alone (0.09 ± 0.01 lesions/10 kb), whereas FHs 74 Int cells showed a decrease in DNA lesion frequency (0.17 ± 0.05 lesions/10 kb) in cells irradiated alone, to undetectable levels in combination treatment (*p* < 0.05 vs. MiaPaCa-2). A potential mechanism for this radioprotection of normal cells and radiosensitization of tumor cells is proposed to be variations in oxidative effects of radiation induced DNA damage in healthy vs. cancerous cells. Finally, this research progressed to a small first-in human phase 1 trial in which intravenous infusion with P-AscH^−^ during radiotherapy revealed treatment safety, supporting the further investigation of ascorbic acid in combination with current chemo-radiotherapy treatments for PDAC [76].

Moreau et al. investigated an unnatural isomer of cannflavin B, a non-cannabinoid, non-psychoactive derivative of *Cannabis sativa L*: FBL-03G [77]. FBL-03G, was found to enhance tumor cell death in combination treatments with radiation on the pancreatic cancer cell lines Panc-02 and Ptf1/p48-Cre (KPC) in vitro and in vivo. More specifically, in vitro exposure to 0, 1, 2, 4 μM of FBL-03G, 24 h prior to radiation exposures of 0, 2 or 4 Gy using 220 kVp energy X-rays, revealed synergistic effects assessed through the clonogenic survival assay. In particular, 4 μM of FBL-03G alone appeared to be more toxic to the cells than 4 Gy. In vivo research investigated tumor volume and animal survival after 100, 200 or 300 µg of FBL-03G and 6 Gy using 220 kVp energy X-rays, revealed significant differences in percentage survival of all combination treatments as compared to 6 Gy radiotherapy alone (*p* < 0.0001). Finally, this research also investigated metastatic regression in distant non-irradiated areas, (known as the abscopal effect) to find that FBL-03G in combination with radiation treatment slowed tumor growth in non-treated areas suggesting further studies into the clinical application of FBL-03G as a radiosensitizer for PDAC [77].

Overall, BFCs have received attention in the literature for chemosensitization research, with a few studies emerging to focus on BFCs as radiosensitizers. Similarly to the chemosensitizer discussed, these pre-clinical data are very much in their infancy, and further investigations are required to report on their potential to sensitize treatments. Natural radio-sensitisers investigated for PDAC are summarized in Table 3.

## 3. Discussion

Novel and natural sources have previously provided successful medical application, including drug approval for ACE inhibitors, antiplatelet drugs, thrombin inhibitors, and chronic painkillers [2]. Moreover, 60% of today’s anticancer drugs originate from natural sources [6]. PDAC continues to challenge therapies with high resistance and complex targeting of the PDAC tumor site, combination treatment attempts generally not being greatly successful. The effect of combining natural compounds with state-of-the-art treatment for PDAC is very much in its infancy and requires further pre-clinical testing to measure their efficacies and de-risk clinical trials. Despite the results discussed above, there is a substantial lack of investigations and subsequent success of natural chemo/radiosensitizers for PDAC in literature, i.e., the majority of this research exists in vitro and are yet to reach clinical stages.

The failure of PDAC treatments at clinical level can be associated with misrepresentative preclinical testing. Developments in treatment testing including new modalities and more effective treatment delivery, i.e., carriers with different functionalities, are being explored to better combat the extremely resistant PDAC TME and protect healthy cells [78,79] However, the majority of the research discussed here utilize traditional preclinical platforms such as 2D cell culture and xenografts, however, there is an absence of reliable and universal measurement approaches to quantify the effects of treatments in these systems and overall, there is a need for more realistic models for PDAC treatment screening. More specifically, 2D cell culture is simplistic and cost effective, however, it does not accurately represent biophysical, biochemical and biomechanical features of the tumor microenvironment associated with in vivo resistance to radiotherapy, such as the desmoplastic response and tumor hypoxia [80,81,82,83]. Xenografts, allow for realistic architecture and more relevant timeframes, however, problems arise with foreign physiology, genetic variation, limited heterogeneity and reduced tumor mutation rates impairing relevance to clinical application [14,80,81,82,83]. Moreover, xenograft models rely on a range of methodologies that are affected by large uncertainties, which contributes to high-risk failure for clinical trials. Three-dimensional (3D) models in the field of tissue engineering are emerging as reliable platforms for radiotherapy treatment screening for PDAC [83]. At the present time, there is a significant lack of 3D models for PDAC as platforms for reliable high-throughput screening of natural treatment in/or without combination treatment approaches. For example, the development of a highly porous polyurethane scaffold system is able to support robust and tunable internal microstructures to allow mimicry of tissue architecture, cell and ECM spatial distribution and realistic environmental gradients as platforms for PDAC cell culture [82,83,84,85,86]. Various other 3D models to mimic a more realistic PDAC microarchitecture and capture more realistic environments as compared to 2D cell culture include spheroid [87,88,89] and hydrogel models [90,91]. Moreover, the 3D toolbox is advancing with patient derived models, and further advancing xenografts such as transgenic mouse models. The application of these 3D models could be a useful tool to improve the efficiency of treatment development to address the issue of misrepresentative preclinical testing, thereafter, the evaluation of natural compounds in a clinical setting is imperative however, these compounds remain firmly in their infancy as anticancer and treatment sensitizers.

## 4. Conclusions

There are a number of natural preclinical compounds emerging for their application in preclinical treatment testing that may hold potential to enhance treatment sensitization, and in turn, reduce treatment resistance and toxicity via lowering treatment dose requirements. This novel research field paves the way for exciting and pioneering anticancer and sensitizers for the treatment of disease, however, the extent of the effectiveness of these products requires careful and universal quantification in reliable preclinical models if they are to progress successfully to the clinic. The exploration of new anticancer and sensitizing compounds is particularly important for PDAC as a cancer of unmet clinical need and a complex radiotherapy history. Two key points are highlighted by this work: (i) the availability of a range of natural compounds for potentially new therapeutic approaches for PDAC, and (ii) potential synergetic impact of natural compounds with advanced chemo- and radio-therapeutic modalities for PDAC.

## Figures and Tables

**Table 1:** Novel and natural anti-cancer targeting compounds for PDAC.

Natural Product/Treatment	Author	Date	Cell Line	Key Findings
Snake Venom	Lucena et al. [49]	2014	Pancreatic Cancer BxPC-3	Integrin antagonists isolated from snake venom induce apoptosis, i.e., 38% and 35% apoptosis/necrosis vs. 96% apoptosis/necrosis after combined exposure (*p* < 0.01).
Plasma	Hattori et al. [56]	2015	Pancreatic Cancer PANC-1 BXPC-3 CaPan-1 MIA PaCa-2	BxPC-3 Pancreatic Stellate Cells hPSC128-SV The first study to investigate antitumor effects of indirect plasma exposure on pancreatic cancer in vitro and in vivo
	Kumar et al. [57]	2018	Pancreatic Cancer MiaPaCa-1	Identified anticancer potential and increased ROS production of plasma treated water and plasma treated media, i.e., down regulation of cell proliferation, cell apoptosis and resistance markers
Ginger and sanguinarine	Prescott et al. [42]	2017	Pancreatic Cancer PANC-1	Suggest anticancer activity of ginger and sanguinarine, and identify them as potential synergistic sonosentisers for PANC-1 cells, i.e., a 6% and 17% increase in cell death as compared to ultrasound alone.
Bee Venom	Celik Uzuner et al. [50]	2019	Pancreatic Cancer AR42J	Black sea bee venom prolonged cytotoxic effects in pancreatic cancer cell in vitro. AR42J cells showed a dose dependent decrease in living cells when treated with 8, 12, 25, 50 and 100 μg/mL for up to 72 h post exposure.
Date Fruit	Al Alwai et al. [4]	2019	Pancreatic Stellate Cells PSC	Ethyl acetate of date fruit significantly reduced PSC’s fibrotic potential. The solvent extracts, ethanol, acetone and ethyl acetate significantly suppressed cell proliferation, i.e., *p* < 0.05, *p* < 0.01 and *p* < 0.001

**Table 2:** Natural chemosensitizers for PDAC.

Natural Product	Author	Date	Cell Line	Key Findings
Oridonin	Bu et al. [61]	2012	Pancreatic Cancer BxPC-2	Oridonin reduced tumor growth (in vivo) and up-regulated MAPK pathways associated with cell cycle arrest and apoptosis, alone and in combination with Gemcitabine as compared to controls (*p* < 0.05)
Trigonelline	Arlt et al. [62]	2014	Pancreatic Cancer MiaPacCa2 PANC-1 Colo357	Trigonelline induced inhibition of Nrf2 activity in combination treatment with Etoposide resulting in apoptotic sensitivity in vitro and in vivo
Melittin	Wang et al. [63]	2017	Pancreatic Cancer SW1990 Capan1 AsPC-1 BxPC-3	Melittin suppressed tumor growth promoting cell apoptosis and cell-cycle arrest and resulted in gemcitabine sensitization via the cholesterol pathway gene clusterin9
Curcumin	Yoshida et al. [65]	2017	Pancreatic Cancer BxPC-3 MiaPaCa2 PANC-1	Curcumin increased gemcitabine toxicity to Gem resistant pancreatic cell lines in vitro and in vivo via PRC2-PVT1-c-Myc axis regulation.
Curcurbitacin D	Sikander et al. [64]	2019	Pancreatic Cancer AsPC-1 BxPC-3 CaPan-1 HPFA-II	Cuc C inhibits expression of key proteins involved in pancreatic cancer cell line chemo-resistance

**Table 3:** Natural radiosensitizers.

Natural Product	Author	Date	Cell Line	Key Findings
Curcumin	Veeraraghavan et al. [73]	2011	Pancreatic Cancer BxPC-3 MiaPaCa-2 PANC-1	Curcumin, neem leaf, raspberry extract inhibited radiation induced NF-κB, and differentially inhibited fractionated radiation and single dose radiation induced genes
Neem leaf extract				
Raspberry extract				
P-AscH^−^ (Vitamin C)	Alexander et al. [76]	2018	Pancreatic Cancer PANC-1 Mia PaCa-2 403	P-AscH^−^, vitamin C induces radio-sensitivity (*p* < 0.05). and radio-protects healthy cells (*p* < 0.05).
Capsaicin and Resveratrol	Vendrely et al. [75]	2019	Pancreatic Cancer PANC-1 Capan-2 BxPC-3 Mia PaCa-2	Capsaicin and resveratrol combination increased sensitivity to radiation in Capan-2 (6 Gy X-ray) as compared to BFCs or radiotherapy alone (*p* < 0.001), and significantly increased ROS production after 6 Gy combination treatment as compared to BFC or radiotherapy alone (*p* < 0.01), significantly reduced tumor volume, (*p* = 0.006 and *p* = 0.001 and inhibited yH2AX production (*p* < 0.05).
FBL-o3G(Cannflavin B)	Moreau et al. [77]	2019	Pancreatic Cancer Panc-o2 Ptf1/p48-Cre (KPC)	FBL-o3G sensitized pancreatic cancer cells to radiotherapy in vitro and in vivo (*p* < 0.0001). The abscopal effect was identified in vivo
Curcumin	Schwarz et al. [74]	2020	Pancreatic Cancer MiaPaCa-2 PANC-1	PANC-1 cells were radiosensitized after 24 h of incubation with 10 (4 Gy: *p* = 0.0048, 6 Gy: *p* = 0.0096) or 12 μm of Curcumin (4 Gy: *p* = 0.0028, 6 Gy *p* = 0.0003, 8 Gy: *p* = 0.00070) Curcumin increased radiation induced apoptosis in both cells lines after 8 Gy exposure (PANC-1: *p* = 0.0174, MiaPaCa-2: *p* = 0.0043)
